# Recent Progress in Silicon Carbide-Based Membranes for Gas Separation

**DOI:** 10.3390/membranes12121255

**Published:** 2022-12-12

**Authors:** Qing Wang, Rongfei Zhou, Toshinori Tsuru

**Affiliations:** 1 School of Energy, Materials and Chemical Engineering, Hefei University, Hefei 230601, China; 2 State Key Laboratory of Materials-Oriented Chemical Engineering, College of Chemical Engineering, Nanjing Tech University, Nanjing 210009, China; 3 Department of Chemical Engineering, Hiroshima University, 1-4-1 Kagamiyama, Higashihiroshima 739-8527, Japan

**Keywords:** silicon carbide membrane, gas separation, precursor-derived ceramics, membrane stability, inorganic membrane, CVD/CVI

## Abstract

The scale of research for developing and applying silicon carbide (SiC) membranes for gas separation has rapidly expanded over the last few decades. Given its importance, this review summarizes the progress on SiC membranes for gas separation by focusing on SiC membrane preparation approaches and their application. The precursor-derived ceramic approaches for preparing SiC membranes include chemical vapor deposition (CVD)/chemical vapor infiltration (CVI) deposition and pyrolysis of polymeric precursor. Generally, SiC membranes formed using the CVD/CVI deposition route have dense structures, making such membranes suitable for small-molecule gas separation. On the contrary, pyrolysis of a polymeric precursor is the most common and promising route for preparing SiC membranes, which includes the steps of precursor selection, coating/shaping, curing for cross-linking, and pyrolysis. Among these steps, the precursor, curing method, and pyrolysis temperature significantly impact the final microstructures and separation performance of membranes. Based on our discussion of these influencing factors, there is now a good understanding of the evolution of membrane microstructures and how to control membrane microstructures according to the application purpose. In addition, the thermal stability, oxidation resistance, hydrothermal stability, and chemical resistance of the SiC membranes are described. Due to their robust advantages and high separation performance, SiC membranes are the most promising candidates for high-temperature gas separation. Overall, this review will provide meaningful insight and guidance for developing SiC membranes and achieving excellent gas separation performance.

## 1. Introduction

With rapid economic development, separation and purification processes are used widely in industrial production and daily life, and their requirements are increasing [1]. However, separating bulk mixtures into pure or purer forms incurs high costs. For example, the current market heavily relies on traditional separation processes and energy-intensive separation methods, such as distillation, which accounts for approximately 10%–15% of global energy consumption [2]. Note that 10 × 10^7^ tons of CO_2_ emissions and USD 4 billion in energy costs could be saved annually if more energy-efficient separation approaches were used in the United States alone. Therefore, ever-increasing industrialization requires more efficient, economical, and sustainable separation and purification technologies. Membrane separation techniques are favored because of their advantages, such as low energy consumption, no need for additives, small footprint, and easy scale-up. Additionally, membrane separation techniques are potential candidates to replace traditional thermal-based separation methods. They are powerful tools for solving critical global problems and for developing new industrial processes required for sustainable industrial growth.

### 1.1. Membrane-Based Gas Separation

Currently, membrane applications mainly involve aqueous media such as wastewater treatment [3], seawater desalination [4], and gaseous media such as air separation, hydrogen production, natural gas purification, and CO_2_ capture [5]. The separation/purification of aqueous media using membranes has been studied extensively and applied successfully in various industries, and membranes were applied for gas separation later than for aqueous media. In 1980, Permea (Monsanto) commercialized Prism membranes for industrial applications in separating hydrogen from purge gas streams of ammonia plants, thus marking the beginning of the first large-scale industrial application of gas separation membranes [6]. Since then, membranes for gas separation have attracted considerable attention from industry and academia, and membrane-based gas separation has exponentially grown.

Membrane-based gas separation does not require phase changes, whereas conventional separation technologies such as adsorption and cryogenic distillation require phase changes with significant energy costs [7]. Generally, permeability (e.g., permeance and flux) and selectivity (or separation factor) are two common and fundamental membrane performance characteristics. The ideal membrane should exhibit high permeability and selectivity. Higher permeability reduces the required membrane area for processing a given amount of gas, thereby reducing the investment cost of membranes. Higher selectivity will result in high-purity gas products. Based on current requirements for advanced membrane technology, achieving the successful application of membranes in gas separation processes not only depends on the high permselectivity of membranes but also on the selection of membrane materials with thermal, hydrothermal, chemical, and mechanical stability [8].

### 1.2. Inorganic Membranes

Membranes can be roughly classified into polymeric and inorganic membranes according to the nature of their fabrication material. Polymeric membranes hold the largest market share and have attracted great interest in commercial-scale applications and academic research. This is mainly due to the economic feasibility of raw materials and convenient membrane manufacture [9]. However, exposing polymers to hydrocarbons or carbon dioxide under high partial pressures could lead to swelling or plasticization, resulting in a significant decrease or loss of separation capacity. Polymeric membranes fall short of the requirements of current advanced membrane technologies as they exhibit a trade-off between permeability and selectivity, with a distinct “upper limit” proposed by Robeson [10,11]. In addition, polymeric membranes cannot withstand aggressive chemical environments and high temperatures.

Compared to polymeric membranes, inorganic membranes have many advantages, such as high chemical, thermal, and mechanical stability. Inorganic membranes have been fabricated from crystalline materials such as zeolites, metal-organic frameworks (MOFs), and amorphous materials. In particular, amorphous inorganic membranes, such as silica, carbon molecular sieves, and silicon carbide-based membranes, also feature better control of pore size and size distribution, as summarized in Table 1. This pore-size control allows for better control of permeability and selectivity.

#### 1.2.1. Silica Membrane

Conventional amorphous silica membranes prepared by sol-gel or chemical vapor deposition (CVD) methods have attracted considerable attention. These membranes are suitable for separating small gas molecules, such as hydrogen and helium, due to their small pore sizes (0.3–0.4 nm) [13,14]. For example, silica membranes used for hydrogen separation (e.g., H_2_/N_2_, H_2_/CO_2_, and H_2_/CH_4_) generally exhibit high H_2_ permeance (10^−6^–10^−8^ mol/(m^2^ s Pa)) with selectivity over hundreds to thousands, which are sufficient to meet the demands of industrial applications [13,15]. However, hydrothermal instability is known to be the biggest issue with silica membranes. When subjected to moist gas streams, water molecules degrade the silica network via the hydrolysis of Si–O–Si bonds, even at low temperatures, resulting in a reduction or loss of membrane separation performance [16,17]. Furthermore, at higher temperatures (e.g., 800 °C), it is easy to cause further sintering of the silica structure to almost full density [18]. These disadvantages limit the application of membranes in most moisture-containing gas or liquid phases. Therefore, in recent decades, considerable efforts have been made to find ways to modify conventional silica membranes, as reported in several reviews [13,14,19,20,21]. Among these efforts, recent progress has been made in developing organosilica membranes that consist of Si–O–Si networks with organic functional groups. Increased hydrothermal stability has been confirmed [13]; however, the temperature used for separation is limited due to the decomposition of organic groups.

#### 1.2.2. Carbon Molecular Sieve (CMS) Membrane

CMS membranes are formed by the pyrolysis of polymer precursor layers under an inert atmosphere or vacuum at elevated temperatures. CMS membranes have rich ultra-microporous structures that distinguish gas pairs with similar molecular size (e.g., C_2_H_4_/C_2_H_6_ separation) [20,22]. In particular, the pore sizes of CMS membranes can be controlled by changing the precursor and pyrolysis parameters [23]. CMS membranes demonstrate satisfactory intrinsic performance for gas separation applications. For example, Ngamou et al. developed a CMS membrane with a thickness of 200 nm on an Al_2_O_3_ support via the pyrolysis of a polyimide precursor at 700 °C [24]. Their CMS membrane exhibited high H_2_ permeability, up to 1.1 × 10^−6^ mol/(m^2^ s Pa), with a selectivity of 24, 130, and 228 for H_2_/CO_2_, H_2_/N_2_, and H_2_/CH_4_, respectively. However, CMS membranes are typically brittle and have variable bimodal pore size distributions that limit their industrial applications [23,25,26]. In addition, the inherently insufficient high-temperature oxidation resistance of CMS membranes limits their application [27].

#### 1.2.3. Silicon Carbide Membrane

**Figure 1:**
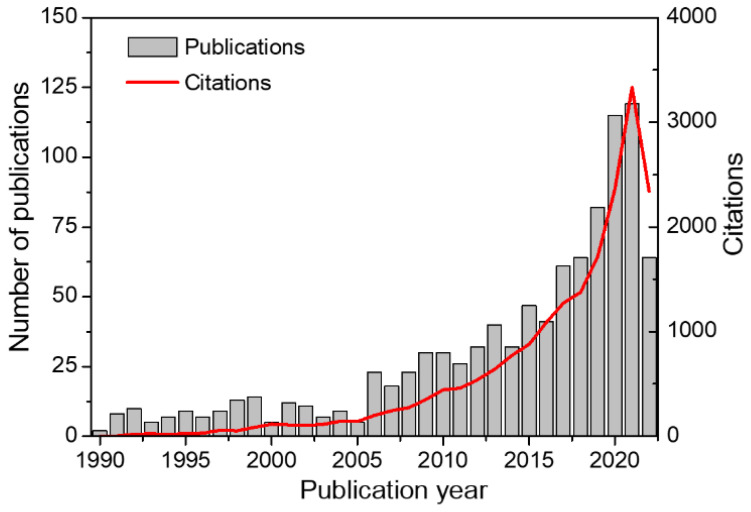
Number of publications and citations on SiC membranes between 1990 and 2022 (Data from Web of Science, accessed on 1 October 2022). The initial search topics were “silicon carbide” or “SiC”, which were subsequently refined for “membrane”.

The production of silicon carbide (SiC)-based and SiC-related materials, including SiC, silicon oxycarbide (SiCO), and silicon carbonitride (SiCN), began more than a century ago (1893) with the heating of a mixture of quartz sand (SiO_2_) and carbon powder [28]. Since then, SiC has rapidly become a promising material in the field of membranes owing to its excellent chemical, mechanical, and hydrothermal stability, high oxidation resistance, thermal shock resistance, and fouling resistance [29,30,31]. In particular, SiC membranes have been adapted for application in harsh environments such as those at high temperatures and when in contact with corrosive chemicals [32,33,34]. SiC membranes have attracted considerable research interest because of the advantages mentioned above. As shown in Figure 1, the number of publications and citations on SiC membranes has grown exponentially over the past 30 years, with the total number of publications reaching over 980 (Data from Web of Science, accessed on 1 October 2022).

To date, only three recent review papers (i.e., Eray et al. [35], Liu et al. [36], and Hotza et al. [37]) on SiC membrane technology have been published. They primarily focus on SiC membranes with large pores used as filters for water treatment and air cleaning. To our knowledge, there is currently no comprehensive review of SiC membranes for gas separation. Because of the growing interest in membrane-based gas separation, this review provides the latest advancements in SiC membrane development and discusses the current applications. Overall, the present review is divided roughly into two main parts. After the introduction, the first part of this review focuses on SiC membrane fabrication methods. The second part discusses the application of SiC membranes and their stability under harsh environments.

## 2. Fabrication of SiC Membranes

**Figure 2:**
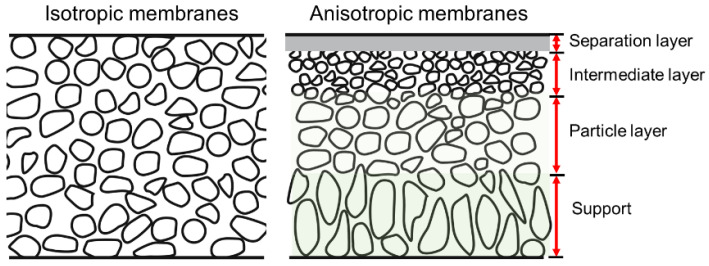
Schematic of isotropic and anisotropic membranes [38].

SiC membranes can be divided according to their morphological construction into symmetrical and asymmetrical types, as shown in Figure 2 [38]. Relatively high permeation fluxes can be obtained via asymmetric membranes, which consist of a support, transition layers (e.g., a particle layer and/or an intermediate layer), and a separation layer. The supports without separation layers, also known as membrane substrates, play an important role in producing defect-free and reproducible separation layers, which in turn play an important role in meeting industrial demand. The membrane substrates should have good characteristics to provide high flux, porosity, and mechanical strength. Currently, materials used as substrates are mainly alumina, mullite, titania, zirconia, SiC, and their composites [35,39]. Among them, SiC is a promising material for fabricating membrane substrates due to its unique properties. Generally, the same SiC substrates can be used as membrane filters, e.g., for microfiltration (MF, 0.05–10 μm), ultrafiltration (UF, 2–50 nm), and nanofiltration (NF, ≤2 nm) [37,40,41]. SiC supports/filters with large pores (>10 μm) are commonly used for air filtering, soot filtering, and catalyst distributors in diesel engines [37,42]. Processing methods for these supports/filters, such as extrusion [43], tape casting [44,45], slip-casting [46], and compression molding [47], have been extensively investigated.

SiC membrane filters usually consist of multiple layers on macroporous supports. Depending on their application purpose, the layers can be designed to generate an upper layer with suitable pore sizes. SiC membrane filters are used mainly in pressure-driven processes, such as MF, UF, and NF, and they have found applications in water treatment, food, and gas cleaning industries. There are a variety of fabrication processes for the fabrication of SiC membrane filters with respect to support characteristics, the range of combinations of raw materials (such as SiC powder compositions, particle sizes, polymer precursors, and sintering additives), deposition techniques, thermal treatment, and applications, as listed in Table 2. The thickness of the top layers ranged from 7 to 125 µm, and the layers were sintered at temperatures up to 2250 °C (Table 2). It is worth noting that the thickness of the top layers (7–19 µm) with transition layers (intermediate layers) was considerably thinner than the top layers directly produced on supports because the transition layers prevent the top-layer solutions from permeating the supports. Additionally, the use of preceramic precursors (e.g., allyl-hydridopolycarbosilane, AHPCS) reduced the thermal treatment temperature significantly to 750 °C, reducing the SiC membrane production cost.

Currently, commercialized SiC membranes are mainly used in MF and UF applications. Regardless of the manufacturer, commercial SiC membranes with high permeability (or flux) and good rejection are used widely in water treatment, especially for MF applications [35,55]. Although the commercial-scale production of SiC membranes is already a mature technology, manufacturing membranes with smaller pore sizes and narrower pore size distributions is a major challenge, such as for water treatment via NF and RO, and particularly for gas separation using microporous SiC membranes. In recent decades, extensive research has been conducted on the preparation of microporous SiC membranes with productive achievements. The subsequent sections focus on the two main precursor/polymer-derived ceramic approaches for fabricating SiC membranes, namely CVD/CVI and the pyrolysis of preceramic precursors.

### 2.1. Chemical Vapor Deposition/Infiltration Route

**Figure 3:**
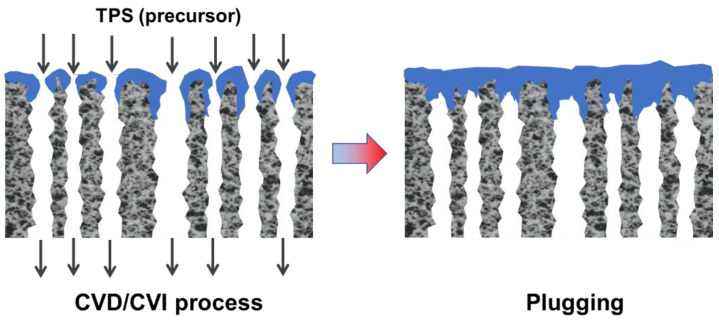
Schematic of SiC membrane preparation via the CVD/CVI deposition process. Adapted from [59] with permission from Elsevier.

**Figure 4:**
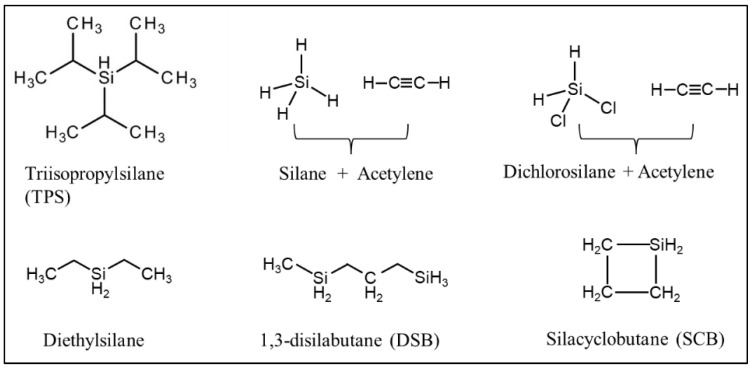
Structures of typical precursors used in CVD/CVI techniques.

CVD/CVI involves exposing a heated substrate to one or more volatile precursors (e.g., organosilica) that react or decompose on the substrate surface (or on the pore walls) to deposit the desired thin film [56,57]. CVD/CVI is a versatile process for manufacturing coatings/films, fibers, powders, and monolithic components [58]. During the formation of SiC membranes, a schematic diagram of the phenomena that could occur via the CVD/CVI deposition process is shown in Figure 3. The precursor (e.g., triisopropylsilane, TPS) is carried by the carrier gas (e.g., Ar) into the pore space of the support, where it reacts to form SiC on the pore surface. SiC gradually shrinks/plugs the pores (especially the macropores) and forms a dense membrane layer [59]. Table 3 lists the representative processing parameters of the SiC membranes obtained by CVD/CVI techniques using the typical precursors shown in Figure 4. It is worth noting that the vast majority of support materials used to fabricate inorganic membranes, as well as SiC membranes, are α-Al_2_O_3_. This is due mainly to the ease of commercialization and high economic competitiveness of α-Al_2_O_3_.

In an early effort, Hong et al. used the SiH_4_/C_2_H_2_/NH_3_ reaction system and α-Al_2_O_3_ supports to fabricate SiC membranes using hot-wall CVD [60]. SiC-Si_3_N_4_ nanoparticles were formed in the gas phase at 1050 °C and deposited in/on the support macropores to form a separation layer with an average pore size of 0.21 μm, which indicates that membrane pore sizes were still in the MF range. Takeda et al. reported the preparation of SiC membranes on γ-Al_2_O_3_-coated tubular α-Al_2_O_3_ supports via CVI using the SiH_2_Cl_2_/C_2_H_2_/H_2_ reaction system at 800–900 °C [62]. The final SiC membranes exhibited H_2_ permeances of 1 × 10^−8^ mol/(m^2^ s Pa) with an H_2_/N_2_ selectivity of 3.36 (Knudsen selectivity of 3.74) at 350 °C. Sea et al. reported pure SiC membranes prepared using triisopropylsilane (TPS) on the macropores of tubular α-Al_2_O_3_ supports using CVD at 700–800 °C, followed by calcination in Ar at 1000 °C [64]. However, these membranes exhibited a Knudsen-type diffusion mechanism without selectivity toward H_2_.

Ciora and Tsotsis et al. were the first to report the fabrication of truly microporous SiC membranes on γ-Al_2_O_3_-coated tubular α-Al_2_O_3_ supports via a CVD/CVI technique using two different precursors (i.e., 1,3-disilabutane (DSB) and TPS) [65]. In their study, the TPS-derived membranes with He permeance ranging from 8.06 × 10^−8^ to 1.72 × 10^−6^ mol/(m^2^ s Pa) and He/N_2_ selectivity ranging from 4 to >100 at 550 °C were hydrothermally stable in the presence of high-pressure (1–3 bar) steam. However, DSB-derived versions with He permeance of 3.5 × 10^−7^ mol/(m^2^ s Pa) and He/N_2_ selectivity of ~55 at 550 °C were not. The preparation procedure of membranes using TPS involved multiple steps that required post-treatment for further structural tailoring at temperatures reaching 1000 °C, which increases the cost. Nagano et al. successfully synthesized a helium-selective SiC membrane on the outer surface of a γ-Al_2_O_3_-coated tubular α-Al_2_O_3_ support by the pyrolysis of polycarbosilane (PCS) at 800 °C under Ar and then modified by CVI using the SiH_2_Cl_2_/C_2_H_2_/H_2_ reaction system [61]. Compared to the original membrane, the membrane modified by CVI exhibited increased He/CO_2_ selectivity from 7.7 to 64 and increased He/H_2_ selectivity from 1.1 to 4.4. However, the He permeance significantly decreased from 8.9 × 10^−7^ to 7.7 × 10^−8^ mol/(m^2^ s Pa) at 600 °C. The authors attributed this to the densification of the Si-C network and the plugging of surface defects during CVI modification.

**Figure 5:**
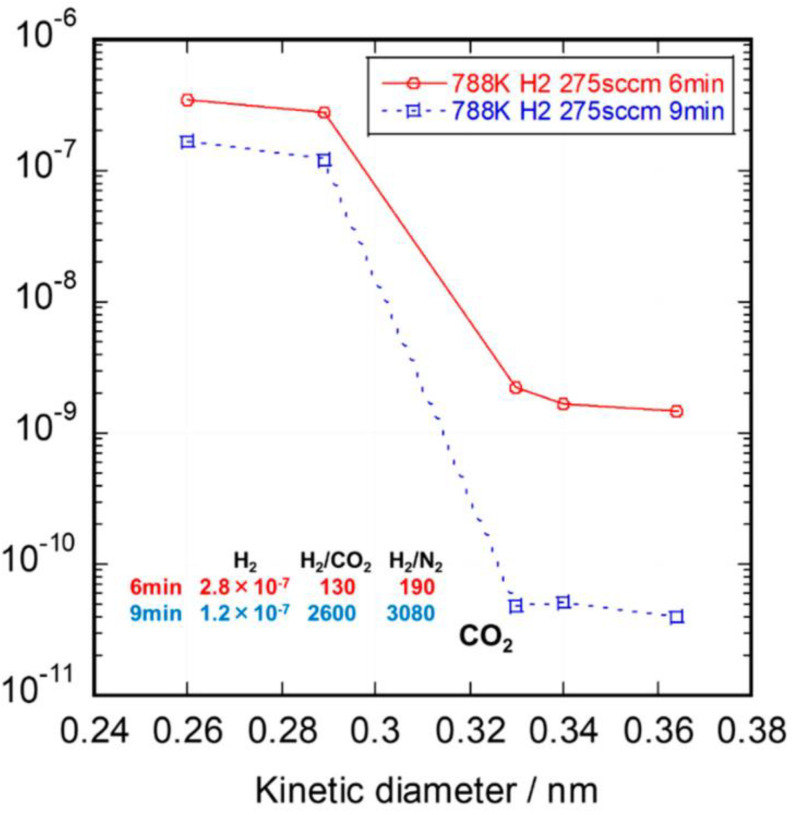
Single-gas permeance of SiC membranes formed by the CVD deposition process. Reprinted from [63] with permission.

Most recently, Nagano et al. synthesized amorphous SiC membranes on Ni-γ- Al_2_O_3_/Al_2_O_3_ supports by counter-diffusion CVD at 515 °C using silacyclobutane and Ar as the carrier gas [63]. As shown in Figure 5, the membrane possessed an H_2_ permeance of 1.2 × 10^−7^ mol/(m^2^ s Pa) and excellent H_2_/CO_2_ selectivity of 2600 at 400 °C at a deposition time of 9 min. The SiC layers were formed within minutes by the CVD/CVI deposition process, which improves the production efficiency of SiC membranes significantly from an industrial point of view. However, as mentioned above, a major issue that remains to be resolved is that SiC membranes formed using CVD/CVI deposition techniques possess dense structures, resulting in low permeability as well as high selectivity suitable for small-molecule gas separation, such as He/H_2_ and H_2_/CO_2_. However, such membranes may not be suitable for separating small-to-mid-sized molecules from larger ones.

### 2.2. Pyrolysis of the Polymeric Precursor Route

**Figure 6:**
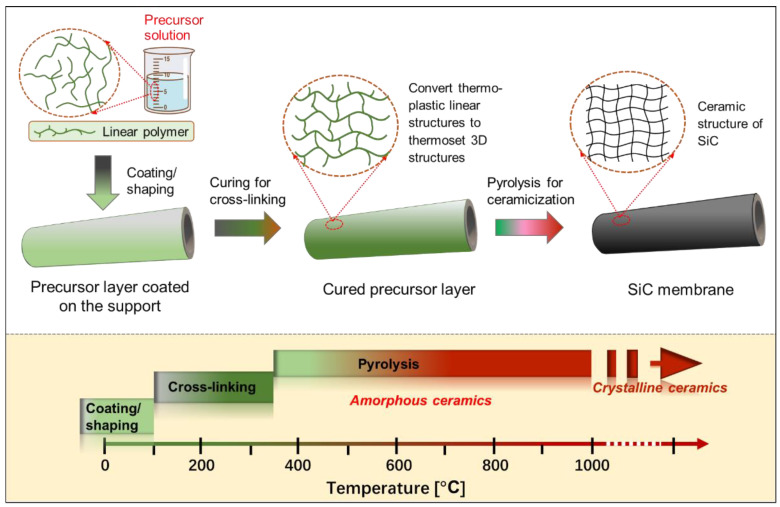
Schematic of pyrolysis for the preceramic precursor route to manufacture SiC membranes. These steps are also temperature dependent and are shown at the bottom.

Another well-known method for preparing SiC membranes is the pyrolysis of coated polymeric precursors. The greatest advantage of this method over the CVD/CVI technique is its processing simplicity, low thermal treatment (pyrolysis) temperatures (≤850 °C), and the fact that production can be carried out either continuously or in a batch process. Furthermore, the preceramic polymeric precursor approach features significant advantages in manufacturing membranes with controlled properties and structures, such as composition, hydrophilic/hydrophobic properties, and pore size, which ultimately affect SiC membrane performance. Figure 6 presents a general schematic of the pyrolysis of the preceramic precursor for manufacturing SiC membranes. In particular, the employed polymeric precursors generally undergo three steps using this route to form SiC membranes, depending on the temperature (these steps are shown at the bottom of Figure 6): coating/shaping, curing for cross-linking, and pyrolysis under an inert atmosphere to complete the polymer-to-ceramic conversion [66]. In this multi-step process, each step is important for obtaining high-quality membranes.

#### 2.2.1. Si-Containing Precursors

**Figure 7:**
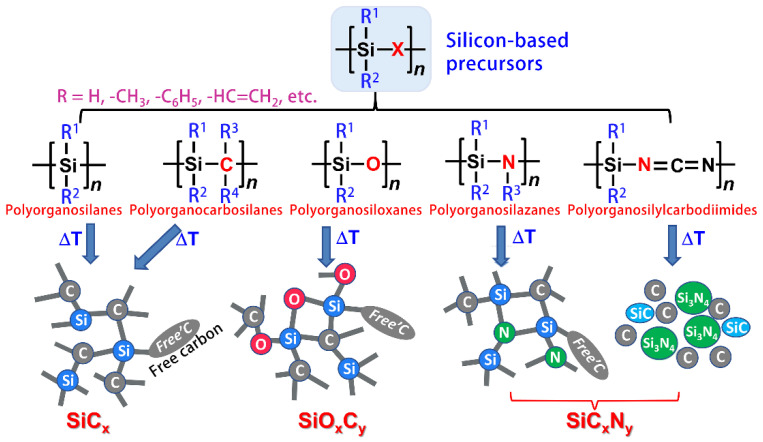
Silicon-containing preceramic polymers and resulting ceramics [67,68,69].

The fabrication of SiC ceramics by the pyrolysis of a polymeric precursor process is strongly influenced by the chemistry and architecture of the Si-based preceramic precursors, their processing routes, and the parameters used for their pyrolysis. Figure 7 presents the general classes of Si-based polymers used as preceramic precursors, polyorganosilanes, polyorganocarbosilanes, polyorganosiloxanes, polyorgano-silazanes, polyorganosilylcarbodiimides, and their corresponding ceramics (e.g., SiC, silicon oxycarbide, and silicon carbonitride) obtained after pyrolysis [67,68,69]. The molecular architecture and type of the preceramic polymer affect not only the composition but also the microstructure of the final produced SiC. Therefore, there is an urgent requirement to develop preceramic polymers with suitable molecular structures, physicochemical properties, and controllable ceramization behaviors [70].

To be suitable for producing SiC ceramic materials, Si-based polymers must meet the following important requirements: (i) the polymers should have a sufficiently high molecular weight to avoid volatilization of components with low molecular weights during subsequent curing and pyrolysis processes; (ii) they should have appropriate rheological characteristics, as well as solubility for the coating/shaping process; (iii) they should have latent reactivity, provided by the presence of specific functional groups (e.g., Si–H, N–H, Si–OH, and Si–CH=CH_2_), which induce cross-linking during the curing process upon exposure to thermal stimuli, chemical stimuli, or irradiation (e.g., UV, electro-beam, and γ-ray).

Continued efforts have been made to develop new strategies to synthesize novel functionalized Si-based polymers inexpensively as precursors and reveal the relationship between their molecular architectures and the properties of final ceramic materials [67]. In the past few decades, several studies have been conducted on the preparation of SiC membranes using different polymeric precursors. Among these studies, polycarbosilanes (PCS) [71,72] and allyl-hydridopolycarbosilane (AHPCS) [73] are the most common polyorganocarbosilanes and will be discussed in the subsequent Section 2.2.3.

PCS-derived SiC ceramics have been investigated extensively and applied to various fields. However, PCS-derived ceramics have drawbacks in their mechanical properties, which cause them to deteriorate at high temperatures [74]. A novel method for the preparation of high-performance SiC ceramics is the introduction of transition metals (e.g., Ti, Al, Zr, and Hf) into SiC ceramics via the pyrolysis of polymetallocarbosilane precursors, which are chemical modifications of PCS. Metal-modified PCS can enhance the comprehensive performance of the final SiC ceramic [75,76].

**Figure 8:**
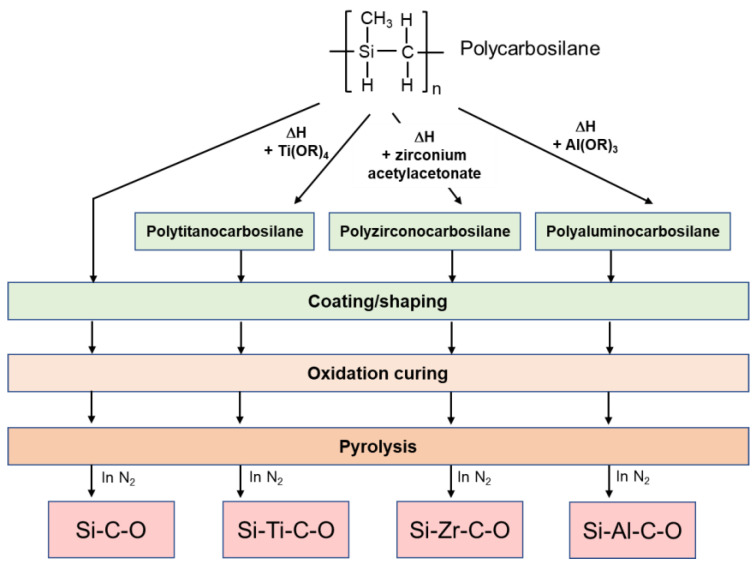
Typical preparation process of metal-containing SiC ceramics derived from polymetallocarbosilane (Ti, Zr, and Al) precursors (with the pure PCS precursor for comparison) [77].

Polymetallocarbosilane is synthesized from PCS by reactions with transition metal alkoxides or metal acetylacetonates, as shown in Figure 8 [74,77,78]. For example, a Ti-incorporated PCS precursor, polytitanocarbosilane (TiPCS), can be synthesized via a reaction of titanium alkoxide (Ti(OR)_4_) with PCS. With the introduction of a small amount of titanium component, the Ti-containing SiC ceramic exhibits enhanced properties such as inhibiting crystalline grain growth of β-SiC up to 1400 °C [78]. Moreover, a Si–Ti–C–O (TiOSiC) fiber, commercialized under the name Tyranno Fiber and marketed by Ube industries LTD. (Japan), has been synthesized using this polymer [79]. The Si–Ti–C–O fiber has many desirable mechanical properties and excellent heat resistance compared to SiC fibers obtained from PCS [74]. The metal-containing SiC ceramics are prepared using a similar process to that of SiC ceramics derived from PCS. The separation properties of membranes derived from the precursors mentioned in this section are discussed later in Section 3.

#### 2.2.2. Coating/Shaping

**Figure 9:**
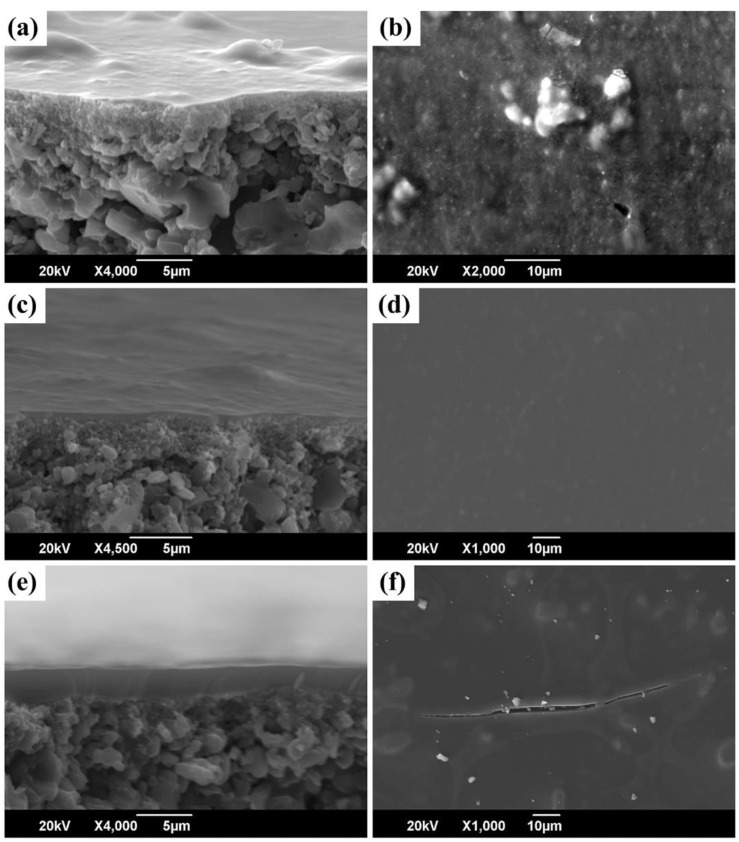
Scanning electron microscopy (SEM) images of cross-sections and surfaces of SiC membranes obtained from different concentrations of coating solutions: (**a**,**b**) 1 wt%; (**c**,**d**) 3 wt%; and (**e**,**f**) 5 wt%. Reprinted from [82] with permission from John Wiley and Sons.

The quality of the coating/shaping depends on the deposition techniques and the solution properties of the precursors. Precursor coating methods mainly include dip-coating [31], wipe-coating [55], casting [54], spin-coating [80], spray-coating [48], and even three-dimensional (3D) printing [81]. Dip-coating and wipe-coating are the most frequently used methods for fabricating membranes because of their relative simplicity. An important factor affecting the coating layers is the viscosity (a rheological characteristic) of the coating solutions, which should be controlled carefully by changing the molecular architectures of the precursors, concentration, and solvent species to achieve coating layers with high quality. This is because the viscosity of the coating solutions could affect the thickness and uniformity of the membrane layers [67,82]. Figure 9 shows the case of the TiPCS precursor using xylene as a solvent [82]. A low concentration (viscosity) of the precursor solution may cause the precursor to penetrate into the macropores of the support or make it difficult to form a continuous membrane layer. However, a high concentration (high viscosity) precursor solution could result in a thicker layer, which can lead to cracking/delamination of the membrane layer during polymer-to-ceramic conversion and increase the likelihood of defect formation.

#### 2.2.3. Curing and Pyrolysis Processes

##### Curing for Cross-Linking

The coated precursor typically requires to be cured at low temperatures (up to 300–400 °C) prior to pyrolysis [71,83], which plays an important role in determining the final quality of SiC ceramic materials, including their microstructural properties. The curing process of polymeric precursors for cross-linking converts the thermoplastic polymers into thermosetting polymers via a series of reactions, such as dehydrogenation and oxidation, which prevents the coating layers/shapes from fusing together during pyrolysis [84]. Additionally, curing is known to promote high ceramic yields of polymeric precursors because cross-linking prevents the volatilization of precursor components with low molecular weight at high temperatures. To date, several curing techniques have been carried out to produce SiC membranes, such as ultraviolet (UV) radiation, electron beam (EB)/γ-ray irradiation [85,86], and conventional thermal treatment under an oxidizing or inert atmosphere [73,87]. These curing techniques induce various condensation (dehydrogenation or demethanization) and addition reactions converting linear polymer networks to 3D polymer networks, which are discussed via PCS and AHPCS precursors in the following sections.

##### Polycarbosilane (PCS) Precursor

Significant progress in the fabrication of SiC ceramic was achieved by the pioneering work of Yajima et al. in the late 1970s via pyrolysis of a PCS precursor (a typical polyorganocarbosilane) with the molecular formula of [–(CH_3_)SiH–CH_2_–]_n_ [88]. Since then, industrial technologies for fiber production have rapidly developed. For example, Ube Industries and Nippon Carbon, two Japanese companies, successfully commercialized PCS-derived SiC fibers [67,77].

**Figure 10:**
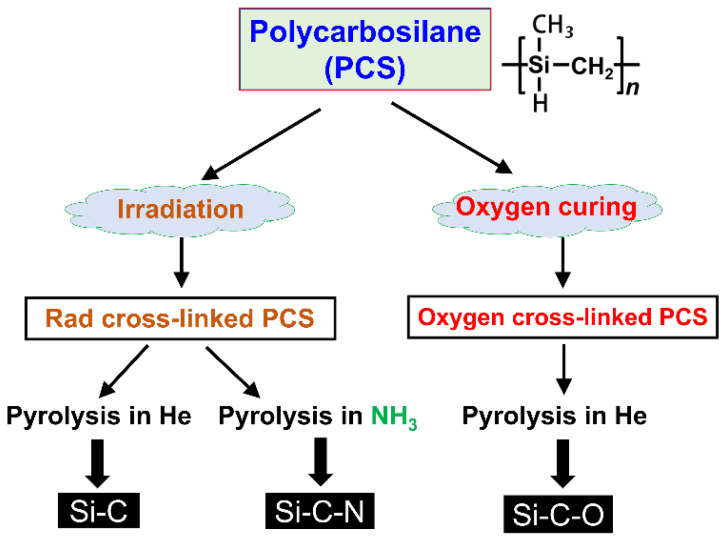
Production process of SiC ceramics under different atmospheres. Adapted from [94] with permission from John Wiley and Sons.

As shown in Figure 10, cross-linking of PCS can be achieved by thermal curing under an air atmosphere or irradiation (UV, γ-ray, or e-beam) under an inert atmosphere [86,87,89,90,91]. Cross-linking of PCS in the presence of oxygen occurs via radical mechanisms: oxidation of Si–H and Si–CH_3_ bonds occurs with the formation of Si–OH, Si–O–Si, and C=O groups [72,92]. However, the cross-linking of PCS in the absence of oxygen involves reactions of Si–H bonds with Si–CH_3_ groups leading to Si–CH_2_–Si linkages [70,93]. Different cross-linking methods and pyrolysis atmospheres produce different types of SiC ceramics [94]. As revealed in Figure 10, three types of SiC ceramics are produced from PCS. The Si–C–O ceramic is produced by oxygen-curing of PCS, and Si–C and Si–N ceramics are produced by radiation cross-linking of PCS. In particular, Si–N ceramic is produced by pyrolysis under an NH_3_ atmosphere [94,95].

##### Allyl-Hydridopolycarbosilane (AHPCS) Precursor

**Figure 11:**
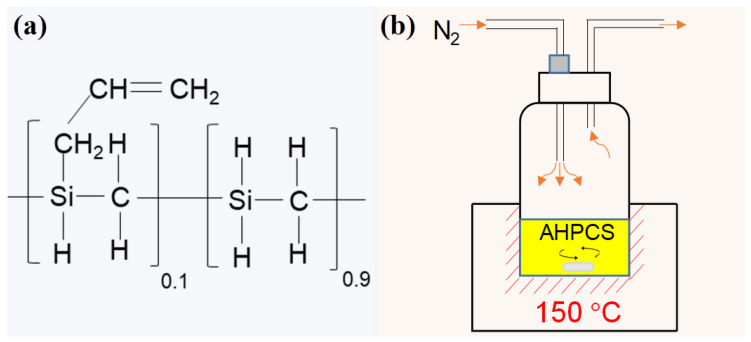
(**a**) Main structure of AHPCS; (**b**) schematic illustration of the thermal curing for pre-cross-linking of AHPCS. Reprinted from [73] with permission from Elsevier.

Allyl-hydridopolycarbosilane (AHPCS, a partially allyl-substituted hydridopolycarbosilane) is a new polymeric precursor. The main structural formula of AHPCS is shown in Figure 11a. The allyl groups (Si–CH_2_–CH=CH_2_) present in AHPCS promote polymer cross-linking when cured under an inert atmosphere, which results in an enhanced ceramic yield [73]. AHPCS-derived SiC membranes are cured for cross-linking under non-oxygen atmospheres, avoiding the introduction of oxygen species, which could enhance the thermal and hydrothermal stability of the final ceramic [35].

**Figure 12:**
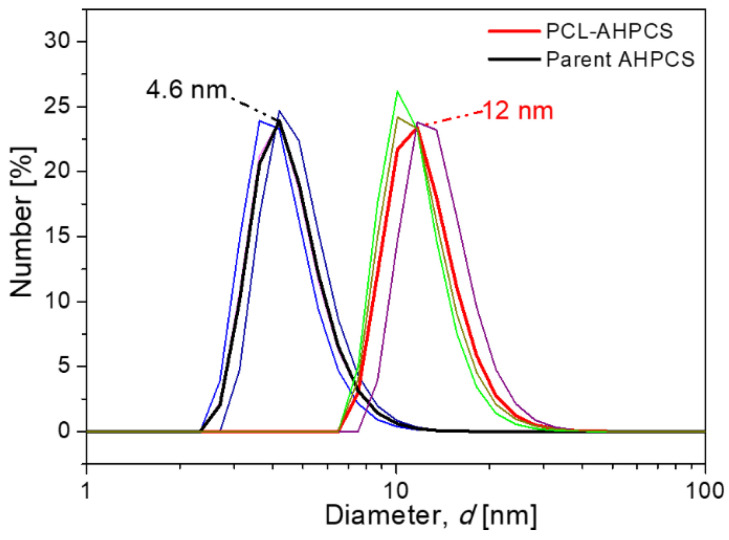
Colloidal size distributions determined by dynamic light scattering for parent AHPCS and pre-cross-linked AHPCS (PCL–AHPCS). The bold lines represent the average of the same sample. Reprinted from [73] with permission from Elsevier.

AHPCS has a liquid form for easy processing and high curing efficiency compared to PCS. AHPCS could undergo curing in the parent solution by thermal treatment for cross-linking. Recently, the author’s group attempted thermal curing of the parent AHPCS solution at a moderate temperature of 150 °C for 2 h under an N_2_ atmosphere for pre-cross-linking (PCL), as illustrated schematically in Figure 11b [73]. The colloidal size of PCL–AHPCS increased from 4.6 nm of the parent AHPCS to 12 nm (as shown in Figure 12), suggesting that the cross-linking behavior increased the polymer size significantly, which could be attributed to the increase in molecular weight through cross-linking. Three major reactions, (1) hydrosilylation/addition, (2) dehydrocoupling, and (3) demethanation, could occur during the thermal cross-linking process, as follows.

≡SiCH_2_CH=CH_2_ + ≡Si-H → ≡SiCH_2_CH_2_CH_2_Si≡ (β-addition) + ≡SiCH(CH_3_)CH_2_Si≡ (α-addition) (1)

≡Si-H + ≡Si-H → ≡Si-Si≡ + H_2_ (2)

≡Si-H + ≡Si-CH_3_ → ≡Si-Si≡ + CH_4_ (3)

It is worth noting that, depending on the reactivity of the functional groups (Si–allyl > Si–H > Si–CH_3_), the first reaction may be the hydrosilylation/addition process. The subsequent cross-linking relies mainly on dehydrocoupling and demethanization reactions that generally occur at higher temperatures.

##### Pyrolysis for Polymer-To-Ceramic Conversion

**Figure 13:**
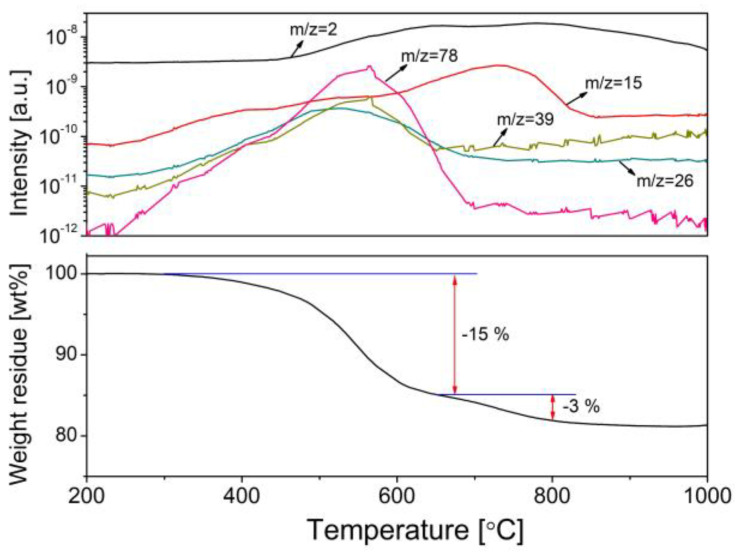
TG–MS curves of Si-containing preceramic polymers (polytitanocarbosilane) under a He atmosphere with a heating rate of 10 °C/min to a level of 1000 °C. Adapted from [55] with permission from Elsevier.

After curing for cross-linking, further thermal treatment at elevated temperatures (usually ≥300 °C) and under an inert atmosphere, i.e., pyrolysis, results in an organic-to-inorganic conversion (from thermoset polymers to amorphous SiC ceramics) [66,73]. This conversion is caused mainly by radicals, condensation (dehydrogenation and demethanization), and rearrangement reactions, which lead to the cleavage of chemical bonds and the formation of new bonds accompanied by the elimination of organic groups and the release of gases, such as H_2_, CH_4_, and C_6_H_6_ (Figure 13) [55]. For most polymeric precursors, the conversion from polymeric precursor to amorphous ceramic is complete at <900 °C, followed by crystallization from the amorphous phase at higher temperatures (>1100 °C) and resulting in phase separation [34,55,71,72,73,82].

**Figure 14:**
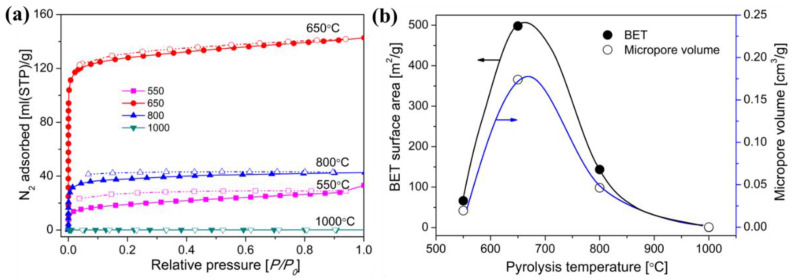
(**a**) N_2_ adsorption–desorption isotherms at 77 K, (**b**) BET surface area and micropore volume (at a relative pressure of *P/P*_0_ = 0.01, pore size ≤ 1 nm) of pyrolyzed precursor (TiPCS) powders. Adapted from [55] with permission from Elsevier.

During the organic-to-inorganic ceramic transformation, the microstructure of polymeric precursors undergoes dramatic changes with increasing pyrolysis temperature [55]. As shown in Figure 14, the microporous properties and pore structure parameters (BET and micropore volume) of the pyrolytic precursor (polytitanocarbosilane, TiPCS) powders were analyzed by N_2_ adsorption–desorption isotherms [55]. The adsorption capacity, micropore volume, and BET surface area increased by firing from 500 °C to 650 °C and then decreased with increasing pyrolysis temperature from 650 °C to 1000 °C. It is worth noting that this is a general conclusion because PCS [72,96], polydimethylsilane (PMS) [97], and AHPCS [73] precursors follow the same trends within a similar pyrolysis temperature range of 300–850 °C. These trends suggest that the decomposition of organic groups generates many micropores at moderate temperatures and the micropores are then narrowed gradually as the pyrolysis temperature increases because of densification and rearrangement reactions.

**Figure 15:**
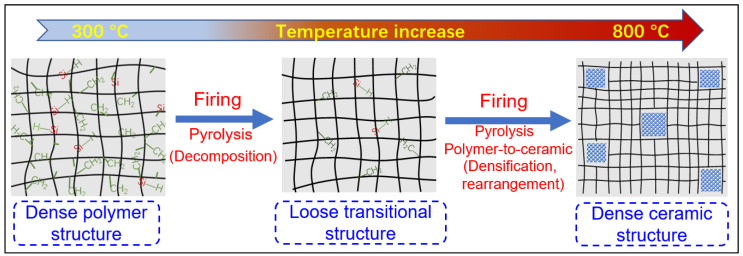
Schematic of the evolution of the network structure for polymeric precursors (e.g., AHPCS) pyrolyzed at different temperatures. Reprinted from [73] with permission from Elsevier.

The evolution of the network structure in polymeric precursors pyrolyzed at various temperatures is schematically illustrated in Figure 15. The evolution of the network structure starts with a dense polymer structure that passes through a loose transitional structure and is then transformed into a relatively denser ceramic structure [55,71,72,73]. It should be noted that this trend would ultimately affect the gas separation performance of membranes, which is discussed later via AHPCS-derived membranes in Section 3.2.

## 3. SiC Membranes for Gas Separation

During the last few decades, important developments in SiC membranes for gas separation have been accomplished. Table 4 lists the SiC membrane-based gas separations and their (potential) applications, which have gained the special attention of researchers. Some gas separation processes will be discussed with the development of SiC membranes in the following paragraphs.

As mentioned earlier, the pyrolysis of polymeric precursors method has been employed as an effective route for forming SiC membranes. Typical PCS [71,72], PMS [97,98], AHPCS [73], and other metal-modified PCS [55,82] precursors have been used to develop SiC membranes for gas separation, which are the main focus of the discussion in this section.

### 3.1. PCS-Derived SiC Membranes

In an early effort, Li et al. [32,33] used polycarbosilane (PCS) as a precursor to prepare SiC (Si–O–C) membranes; PCS was coated on α-Al_2_O_3_ tubular supports, cured at 200 °C in air, and pyrolyzed at high temperatures using polystyrene (PS) as a pore former. A membrane prepared using the single component of PCS pyrolyzed at 950 °C revealed H_2_ permeance of around 1 × 10^−8^ mol/(m^2^ s Pa) and an H_2_/N_2_ selectivity of 18–63 at a permeation temperature of 500 °C [32]. After adding 1% PS to the PCS, the membrane showed an H_2_ permeance of 4 × 10^−8^ mol/(m^2^ s Pa) with an H_2_/N_2_ selectivity of 20 [33]. When 5% PS was added to the PCS, the H_2_ permeance effectively increased to ~9 × 10^−8^ mol/(m^2^ s Pa) with an H_2_/N_2_ selectivity of 13 at the same temperature of 500 °C [32]. This result indicates that the pore-forming agent PS can increase the pore size of the membrane, thereby increasing the permeance and reducing the membrane selectivity.

**Figure 16:**
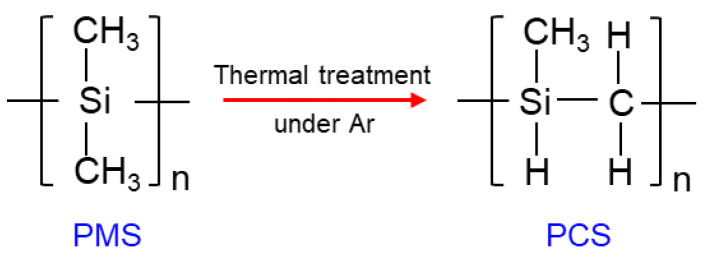
Schematic illustration of the conversion reaction of PMS to PCS.

Lee and Tsai prepared SiC membranes by the pyrolysis of polydimethylsilane (PMS) [97,98,99]. In their work, the PMS layers deposited on the supports were first reacted at 460 °C under an Ar atmosphere for 14 h to allow the methylene group of the PMS to insert into its backbone to convert to the PCS precursor (Figure 16) [98,100].

Next, the coated supports were air cured for cross-linking at 200 °C (or 250 °C) for 1 h and pyrolyzed at 300–950 °C for 1 h in Ar to obtain SiC membranes. One membrane pyrolyzed at 600 °C demonstrated the highest separation performance, exhibiting an H_2_ permeance of ~2.7 × 10^−9^ mol/(m^2^ s Pa), an ideal H_2_/N_2_ selectivity of 20, and an H_2_/*i*-C_4_H_10_ selectivity of ~80 at 200 °C [97]. However, permeance and selectivity were significantly reduced for membranes prepared at pyrolysis temperatures above 600 °C, which was attributed to the sintering (or densification) of the membrane micropores at high temperatures. Further studies on the hydrothermal stability of the membranes revealed that the permselectivity of the PMS-derived membranes was destroyed at 300–500 °C under an H_2_O partial pressure of 0.23 mbar. This demonstrated the poor hydrothermal stability of membranes derived from PMS [99]. The possible reason for this result is that their SiC membranes introduced excess oxygen elements and formed a large number of Si–O–Si bonds, rather than Si–C, during the air curing process for cross-linking.

**Figure 17:**
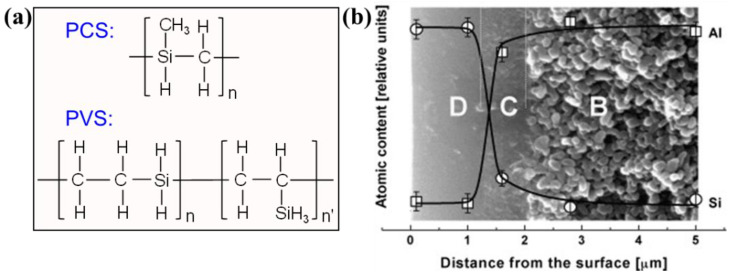
(**a**) Chemical structures of PCS and PVS; (**b**) SEM image of the cross-section of PCS/PVS-derived membranes (the inset shows the atomic content curves of Al and Si throughout the sample cross section measured by energy dispersive X-ray spectroscopy; B: α-alumina, C: γ-alumina, D: SiC membrane layer, 1.25 μm) (adapted from [101] with permission from Elsevier).

Subsequently, Wach et al. [85,101] prepared SiC (Si–O–C) membranes via PCS and polyvinylsilane (PVS) precursor blends (the chemical structures are represented in Figure 17a). The blended precursors were spin-coated on porous γ-Al_2_O_3_-coated α-Al_2_O_3_ plates and cured for cross-linking via EB/γ-irradiation in the presence of oxygen. This was followed by pyrolysis at 850 °C to obtain SiC membranes with a thickness of 1.25 μm (Figure 17b). These membranes achieved an H_2_ permeance range of 10^−10^–10^−8^ mol/(m^2^ s Pa) with H_2_/N_2_ selectivity of 254 at 250 °C [101]. The PVS-added versions had higher H_2_/N_2_ selectivity but lower H_2_ permeance than the pure PCS-derived membranes. This was attributed to the small pore sizes obtained from their dense cross-linked structures. The addition of PVS with a high concentration of active functional groups (Si–H) to PCS promotes cross-linking reactions. Densely cross-linked networks are formed, particularly as more active free radical reactions under EB irradiation further enhance cross-linking [101,102]. In addition, high-viscosity PVS has plasticizing properties enabling the precursor layer to form smoother coating layers on the substrates during solvent evaporation and preventing crack and pinhole formation.

Suda et al. [103,104] prepared SiC membranes with separation layer thicknesses of less than 1 μm by dip-coating PCS on microporous α-Al_2_O_3_ tubes (average pore size: 150 nm). Their cross-linked PCS-derived SiC membrane (thermal cross-linked at 200 °C for 10 h and pyrolyzed at 700 °C for 2 h) exhibited H_2_ permeance of 1–3 × 10^−8^ mol/(m^2^ s Pa) at 100 °C, which was ~5–10 times higher than the corresponding non-cross-linked membranes. These membranes also retained high H_2_/N_2_ selectivity (90–150). This is because SiC membranes derived from cross-linked PCS have a larger number of smaller micropores than non-cross-linked PCS [103].

In previous studies, most SiC membranes have shown a low level of gas permeance due to their dense structures with smaller pores. For example, the H_2_ permeance of these membranes was ~10^−9^–10^−8^ mol/(m^2^ s Pa) but H_2_/N_2_ selectivity greatly varied. Based on these earlier efforts, the authors’ group proposed for the first time that the microstructure and permeation properties of PCS-derived membranes could be tuned via a curing process [72]. The microstructural variations of PCS-derived membranes were investigated systematically at air curing temperatures of 150–350 °C and pyrolysis at 750 °C. Air curing for cross-linking increased the thermal stability of PCS and PCS-derived ceramic structures compared to the uncured version. The elemental composition of the PCS-derived final ceramic was positively correlated with the air-cured precursor. The elemental composition could be tailored precisely via the degree of air-curing-induced oxidation of Si–H groups. Different ceramic compositions generally feature different structural properties that impart different separation properties to membranes. However, variations of the microporous structures of the final ceramics and the gas permeation properties of membranes as a function of the curing temperature suggest that either excessive or insufficient cross-linking using air curing tends to cause the micropores to collapse or disappear at high temperatures. PCS-derived ceramic/membranes air cured at 250 °C featured a stable and uniform microporous structure attributed to appropriately cross-linked networks.

**Figure 18:**
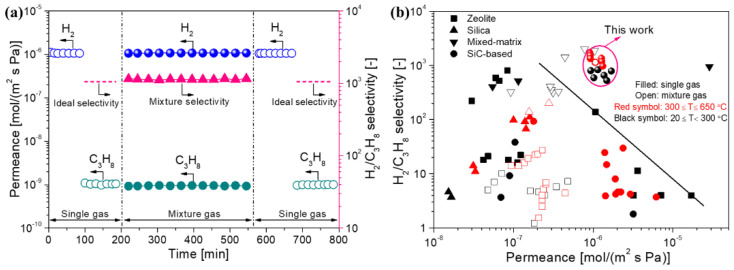
(**a**) Permeance and selectivity for H_2_/C_3_H_8_ through PCS-derived membranes as a function of the test time at 500 °C (mixture: 50/50, feed pressure: 250 kPa (abs.), permeate pressure: 100 kPa (abs.)); (**b**) comparison of separation performance of various types of membranes for H_2_/C_3_H_8_ at 20 °C–650 °C. Adapted from [72] with permission from Elsevier.

**Figure 19:**
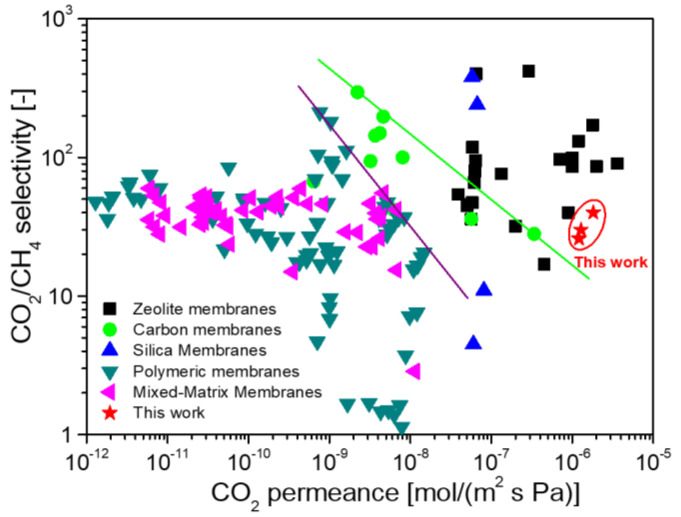
Comparison of separation performance of various types of membranes for CO_2_/CH_4_ at or around room temperature. Reprinted from [72] with permission from Elsevier.

The appropriately cross-linked PCS-derived membranes had excellent gas separation performance, such as an H_2_/N_2_ selectivity up to 31 with high H_2_ permeance of 1–2 × 10^−6^ mol/(m^2^ s Pa) at 500 °C [72]. The membranes were also evaluated for H_2_/C_3_H_8_ mixed gas separation at a high temperature of 500 °C, as shown in Figure 18a. Interestingly, the separation performance (including permeance and selectivity) in the H_2_/C_3_H_8_ mixture was approximately the same as that in single gases, suggesting that the separation mechanism could be dominated by molecular sieving. Figure 18b compares the separation performance for H_2_/C_3_H_8_ in various membranes. The PCS-derived membranes exhibited superior H_2_ permeance of 1–2 × 10^−6^ mol/(m^2^ s Pa) with H_2_/C_3_H_8_ selectivity up to 1740, which favorably compares with zeolite, silica, and other SiC membranes across a wide range of temperatures (20–650 °C). The excellent separation performance of H_2_/C_3_H_8_ indicates that the PCS-derived SiC membranes are promising in membrane reactors for the high-temperature dehydrogenation of propane. It is worth noting that these membranes also have satisfactory separation performance for CO_2_/CH_4_ [72]. As shown in Figure 19, PCS-derived membranes exhibited superior CO_2_ permeance of 1.8 × 10^−6^ mol/(m^2^ s Pa) with moderate CO_2_/CH_4_ selectivity of 40, which far surpasses the upper bounds of the polymer membranes, carbon membranes, or mixed-matrix membranes. It also demonstrates that PCS-derived membranes provide a competitive choice for membrane materials suitable for CO_2_/CH_4_ separation.

### 3.2. AHPCS-Derived SiC Membranes

As mentioned earlier, AHPCS is a new polymeric precursor with a liquid form for easy processing. This precursor is highly efficient in curing under inert atmospheres, rather than oxygen-containing atmospheres, compared to PCS. Tsotsis’ group [65,105] used AHPCS as a polymeric precursor and deposited it onto tubular SiC supports by combining slip-casting and dip-coating techniques. This was followed by pyrolysis at 750 °C to produce SiC membranes. These membranes exhibited an H_2_ (or He) permeance in the range of 10^−9^–10^−8^ mol/(m^2^ s Pa) at 200 °C, an H_2_/CO_2_ selectivity in the range of 42–96 [105], an H_2_/CH_4_ selectivity in the range of 29–78 [105], a He/N_2_ selectivity of ~20 [65], and a He/Ar selectivity of 147 [105]. In their other paper [31], SiC membranes were prepared on highly permeable SiC supports using AHPCS as a precursor. That membrane showed a He/Ar selectivity of up to 1100 with He permeance of ~9 × 10^−9^ mol/(m^2^ s Pa) at 200 °C. The significant improvement in He/Ar selectivity indicated that the pore sizes of the membranes derived from AHPCS could be tuned.

**Figure 20:**
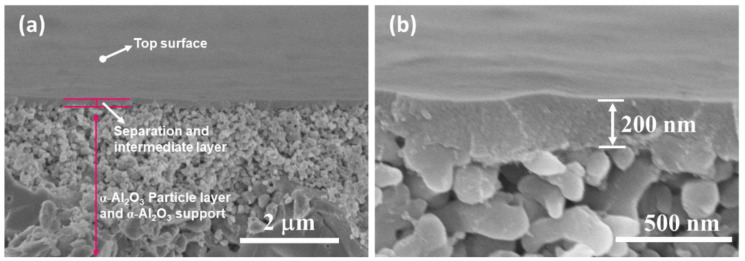
SEM images of the top surface and cross-section of pre-cross-linked AHPCS-derived membranes: (**a**) low and (**b**) high magnification. Reprinted from [73] with permission from Elsevier.

**Figure 21:**
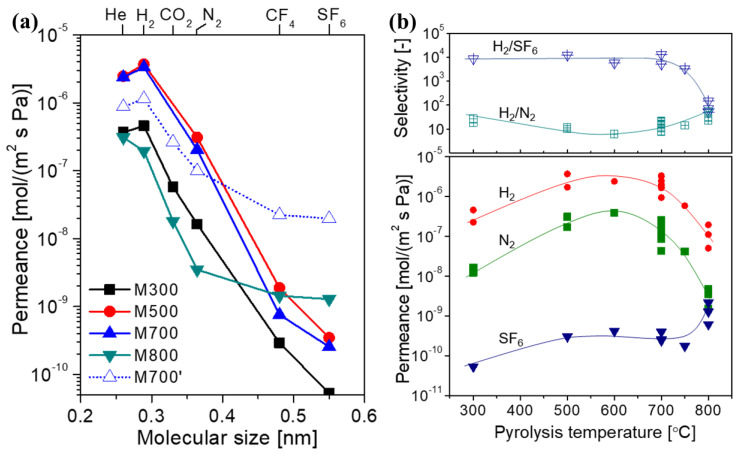
(**a**) Single-gas permeance at 200 °C for AHPCS-derived membranes pyrolyzed at different temperatures (M700 was derived from the parent AHPCS solution, while M700′ was derived from the pre-cross-linked AHPCS solution); (**b**) relationship between the gas permeation performance and pyrolysis temperature. Adapted from [73] with permission from Elsevier.

In our most recent paper [73], the parent AHPCS solution was cured thermally for pre-cross-linking (PCL) at a low temperature of 150 °C for 2 h under an N_2_ atmosphere (Figure 11b) for the first time, resulting in a significant increase in the colloidal size of AHPCS from 4.6 to 12 nm (Figure 12). The advantages of PCL are as follows. The coated precursor on the substrates does not need to be cured further for cross-linking, which significantly saves SiC membrane manufacturing time and improves production efficiency. In addition, the coating solution of pre-cross-linked AHPCS with a macromolecular size effectively reduces penetration into the substrates and improves the gas permeability, which is also beneficial for obtaining defect-free membranes. These advantages were confirmed by SEM images of the microtopography (Figure 20) and the membrane gas permeation performance (Figure 21a). A thin, continuous, and crack-free separation layer was deposited onto a SiO_2_–ZrO_2_–coated tubular α-Al_2_O_3_ support, which clearly showed that the top layer barely penetrated the α-Al_2_O_3_ particle layer. Additionally, an M700 membrane derived from the pre-cross-linked solution exhibited higher permselectivity than an M700′ derived from the parent AHPCS solution.

Furthermore, the effects of the pyrolysis temperature on the gas separation performance of SiC membranes were investigated systematically using the AHPCS precursor, as shown in Figure 21. As the pyrolysis temperature was increased from 300 °C to 800 °C, the permeance of small molecules (e.g., He, H_2_, and N_2_) first increased and then decreased. In contrast, the selectivity of small molecules over large molecules (e.g., N_2_, CF_4_, and SF_6_) exhibited an opposite trend. This indicates that SiC membranes prepared at low temperatures (e.g., T ≤ 350 °C) feature dense polymer structures with small pores, resulting in high selectivity but low permeability due to the numerous organic groups in the pores. When the pyrolysis temperature was increased to a moderate level (e.g., 350 °C < T < 700 °C), most organic groups in the backbone network were removed by decomposition reactions, forming a loose transition structure with larger pores. With the further increase in pyrolysis temperature (e.g., T ≥ 700 °C), these transitional structures shrank through rearrangement and densification reactions to form relatively denser ceramic structures with narrow pores. This is a general conclusion, such as for membranes derived from the PCS [72,96] and PMS [97] precursors following the same trend (Figure 15).

### 3.3. Other Element-Doped SiC Membranes

**Figure 22:**
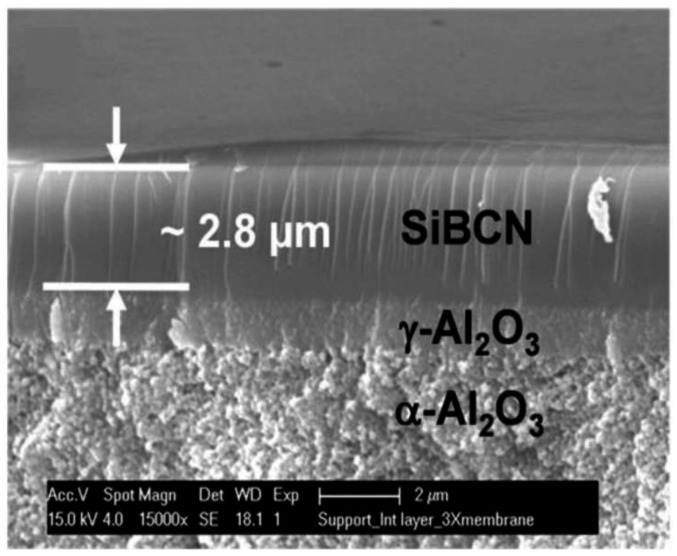
Cross section of a multilayer Si–B–C–N/γ-Al_2_O_3_/α-Al_2_O_3_ membrane. Adapted from [107] with permission from John Wiley and Sons.

PCS, the most typical polymeric precursor, is used widely in SiC ceramic applications. However, PCS-derived ceramic materials still suffer from some disadvantages, such as the easy formation of dense structures at high fabrication temperatures or deterioration at high service temperatures. Metallic elements, such as Al, Ti, and Zr, and nonmetallic elements could improve the heat resistance, structure stability, and electrical properties of the final SiC ceramic product, as well as the SiC membranes [55,106]. However, few studies focused on these element-doped SiC membranes for molecular separation. Prasad et al. reported a B-containing multilayer Si–B–C–N/γ-Al_2_O_3_/α-Al_2_O_3_ membrane prepared at 800 °C with a thickness of ~2.8 μm, as shown in Figure 22 [107]. The membrane exhibited an H_2_/CO selectivity of ~10.5 with an H_2_ permeance of 1.05 × 10^−8^ mol/(m^2^ s Pa) at 440 °C. The thermal stability of this material up to 1100–1500 °C was promising for H_2_ purification at high temperatures.

The authors’ group was the first to report the fabrication of titanium (Ti)-doped sub-nanoporous SiC membranes derived from polytitanocarbosilane (TiPCS) [68,82]. The TiPCS precursor was synthesized by a reaction of titanium alkoxide with PCS. Table 5 lists the BET surface areas and micropore volumes of the pyrolyzed powders derived from PCS and TiPCS as a function of the pyrolysis temperature and under the same preparation conditions. When the pyrolysis temperature was higher than 650 °C, the BET and micropore volume values of the TiPCS samples were significantly higher than those of the PCS version. For example, the BET surface area of PCS decreased sharply and fell to less than 1 m^2^/g, with a micropore volume of zero at 750 °C. This was attributed to pore shrinkage and severe densification at high pyrolysis temperatures. In contrast, the TiPCS-derived powders maintained their microporosity, even at a higher pyrolysis temperature of 800 °C, which could be attributed to the longer Ti–O bond length (~1.85–2.10 Å) in the network structures compared to that of Si–O (~1.570–1.639 Å) [108,109]. This result demonstrates that the Ti component in PCS effectively inhibits and/or reduces the densification of the network structures at high temperatures, which would ultimately affect the separation performance of the TiPCS-derived membranes.

**Figure 23:**
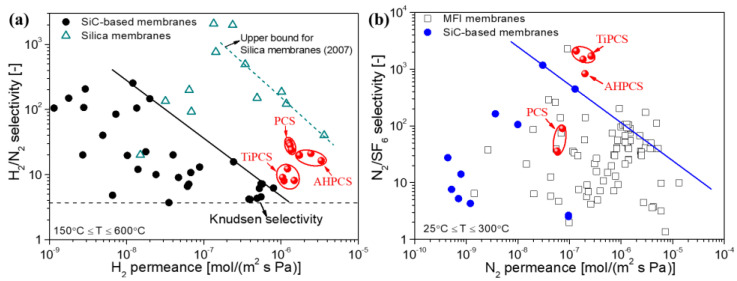
Comparison of the selectivities for (**a**) H_2_/N_2_ and (**b**) N_2_/SF_6_ by various membranes [72,73,82].

Figure 23 summarizes the performance of SiC ceramic membranes (together with PCS [71,72], AHPCS [73], and TiPCS [55,82] derived membranes with similar separation layers in thicknesses reported by the authors’ group) for small/medium molecules (H_2_/N_2_) and medium/large molecules (N_2_/SF_6_) separation. It is worth pointing out that SF_6_ is a gas with the largest molecular size and is often used to evaluate the presence of large pores. However, N_2_/SF_6_ is usually used as a benchmark to evaluate the separation performance of mid to large-sized molecules [110].

Obviously, the separation performance or pore sizes of SiC membranes can be tuned by the preparation conditions (such as curing, precursor species, and element doping) and according to the application purposes. The TiPCS-derived membrane exhibited a low H_2_/N_2_ selectivity (around 10) due to the nature of relatively large sub-nanopores. However, it had superior N_2_/SF_6_ permselectivity to other SiC membranes and MFI membranes (pore size ≈ 0.55 nm [111]). This indicates that the TiPCS-derived membrane is suitable for the separation of mid to large-size molecules, such as MeOH/toluene and MeOH/MTBE, via pervaporation (PV) [82]. Most PCS-derived membranes exhibit higher separation performance for H_2_/N_2_ (as well as H_2_/C_3_H_8_) but moderate permselectivity for N_2_/SF_6_ due to their dense ceramic structures. However, AHPCS (Figure 11a) has a larger allylic branched structure relative to the PCS precursor (Figure 17a), which possibly induces moderately dense networks (i.e., medium-sized pores) to endow moderate separation performance for H_2_/N_2_ and N_2_/SF_6_. Robust SiC membranes with different pore sizes would have many new applications. Therefore, the pore size control of SiC membranes in the field of molecule separation has attracted considerable research interest.

## 4. Stability under Harsh Conditions

### 4.1. Thermal Stability and Oxidation Resistance

**Figure 24:**
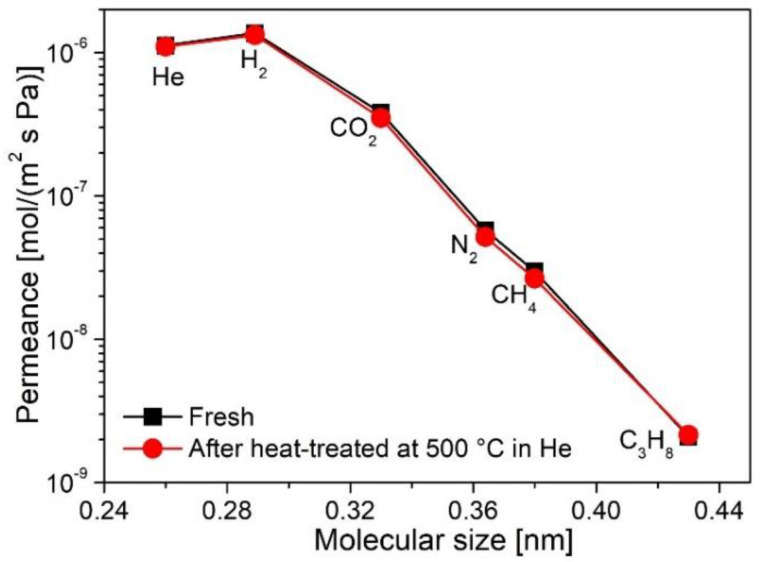
Single-gas permeance at 200 °C as a function of molecular size for a PCS-derived membrane before and after thermal stability testing at 500 °C under a He atmosphere for 12 h. Adapted from [72] with permission from Elsevier.

A fundamental issue and ongoing challenge for the widespread application of membranes is their stability, particularly under harsh conditions (such as high temperatures, the presence of steam, and resistance to acids/alkalis). Many designed applications of SiC membranes are usually carried out at high temperatures and in the presence of air and/or water-containing gas streams. Therefore, these membranes should have high thermal stability, oxidation resistance, and hydrothermal stability. Many studies have shown that SiC membranes have robust thermal stability under inert atmospheres because of the SiC structure generated by pyrolysis at high temperatures under inert atmospheres. For example, Figure 24 shows the molecular size dependence of single-gas permeance at 200 °C for a PCS-derived membrane before and after thermal stability testing at 500 °C under He flow for 12 h [72]. Clearly, no appreciable change in separation performance was observed for PCS-derived SiC membranes, which indicates a high level of thermal stability for SiC membranes.

**Figure 25:**
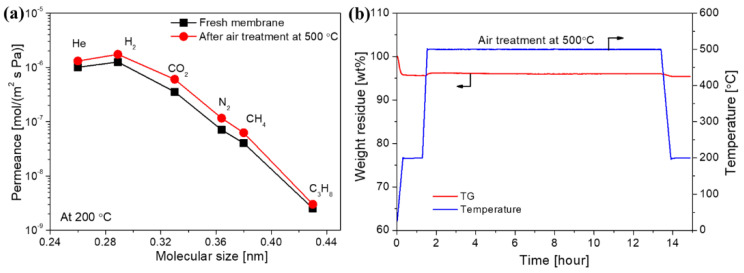
(**a**) Single-gas permeance at 200 °C for a PCS-derived membrane before and after air treatment at 500 °C; (**b**) time-course changes in the weight of PCS-derived SiC powder in air at 500 °C following 12 h. The powder was prepared under the same conditions as the membrane. Adapted from [72] with permission from Elsevier.

However, most carbon-based membranes have insufficient oxidation resistance due to the presence of large amounts of free carbon. Free carbon is also believed to be present in SiC materials [112,113,114]. Figure 25a exhibits the single-gas permeance of a PCS-derived membrane before and after air treatment at 500 °C [72]. After the air treatment, the gas permeance of the membrane increased slightly, but there was little change in selectivity. This is because the removal of free carbon could reduce the steric resistance in the pores and improve the gas permeability, while the selectivity was maintained due to the high stability of the SiC skeleton structure. Therefore, the SiC membrane has good oxidation resistance, as confirmed by the thermogravimetric (TG) analysis shown in Figure 25b. It is worth noting that the AHPCS-derived SiC membrane also exhibited similar results after air treatment at 500 °C for 24 h, which again confirmed the high oxidation resistance of SiC membranes [73].

### 4.2. Hydrothermal Stability

The hydrothermal stability of membranes at high temperatures is an important property. However, only a limited number of papers have reported the hydrothermal stability of SiC membranes. The hydrothermal stability of SiC membranes is related to the membrane preparation conditions. SiC membranes derived from AHPCS were exposed to an equimolar mixture of steam and He at 200 °C for 14 days [105]. The He permeance of the membrane initially decreased and then reached a steady state under these conditions. However, the permeance of He did not recover after the membrane was dried for 48 h. Nevertheless, the SiC membrane maintained high permselectivity after a steam treatment. The reason for this phenomenon was that Si–O might exist on the surface of the membrane and/or SiC might be oxidized by water vapor at high temperatures, which generates Si–OH groups and increases the permeation resistance. Furthermore, the effect of steam on the membrane is limited to the surface region rather than causing bulk transformations of the SiC material [105].

Recently, the authors’ group reported a Ti-doped SiC membrane derived from TiPCS for equimolar H_2_O/N_2_ mixture separation at 300 °C [82]. During the initial 3.5 h of steam exposure, the permeance of H_2_O slightly increased while the permeance of N_2_ significantly decreased (by 70%), after which a steady state was reached. As a result, the separation factor of H_2_O/N_2_ was increased significantly, from 14 to 40, along with the H_2_O permeance of 1.64 × 10^−6^ mol/(m^2^ s Pa). This could be attributed to the gradual generation of Si–OH groups on the surface of the membrane pores, which increase the affinity and permeability to small-sized H_2_O molecules (0.296 nm). However, the permeation of large-sized N_2_ molecules (0.36 nm) through the pores was inhibited by Si–OH groups and/or adsorbed H_2_O [115,116]. After drying the membrane at 500 °C for 3 h, the N_2_ permeance was almost recovered, and the single-gas permeation performance of the membrane was similar to that of a fresh membrane. This indicates that -OH groups of membrane surface could be removed during the drying process, but the pore structure of the membrane was not changed. The above results demonstrate that the SiC membrane has good hydrothermal stability and SiC membranes maintain high separation performance, even under harsh steam conditions. Therefore, the SiC membrane is one of the most promising candidates for gas dehumidification in membrane contactor systems and membrane reactors in the water–gas shift reaction.

### 4.3. Chemical Resistance and Membrane Reactors

**Figure 26:**
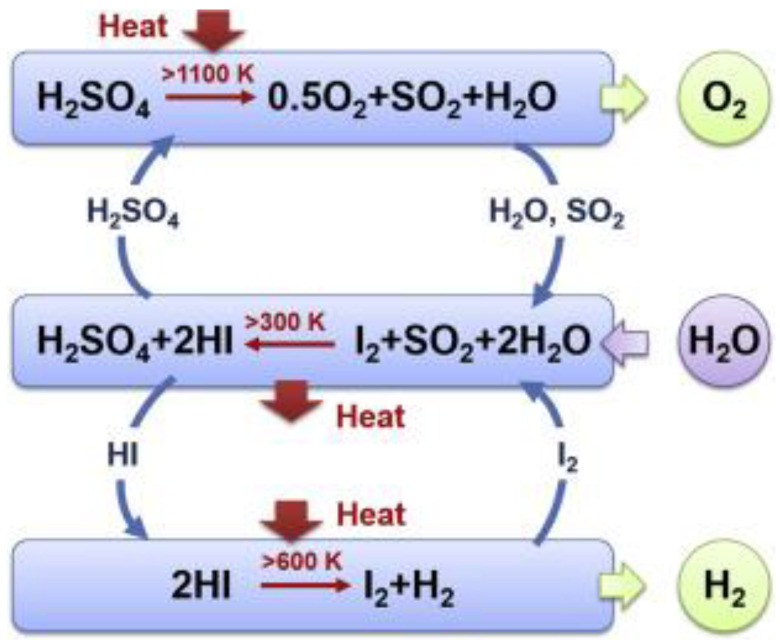
Schematic of the iodine–sulfur thermochemical water-splitting cycle. Reprinted from [117] with permission from Elsevier.

It has been discussed that SiC membranes have good thermal stability, oxidation resistance, and hydrothermal stability. Their chemical resistance is discussed through a membrane reactor. The water-splitting iodine–sulfur (IS) process has been studied extensively as a sustainable technology. The IS process requires a much lower temperature for the net production of O_2_ and H_2_ than the direct thermal decomposition of H_2_O [117]. As shown in Figure 26, the IS process primarily comprises three chemical reactions involving the decomposition of H_2_SO_4_ and HI, followed by the regeneration of these reagents via the Bunsen reaction [118]. However, the decomposition of H_2_SO_4_ in this process must be performed at high temperatures (above 827 °C), which limits the wide application of IS for H_2_ production in the industry due to strict equipment requirements. Therefore, it is a promising strategy to extract O_2_ from the sulfuric acid decomposition reaction through a membrane reactor to reduce the reaction temperature under the effect of equilibrium shift. 

**Figure 27:**
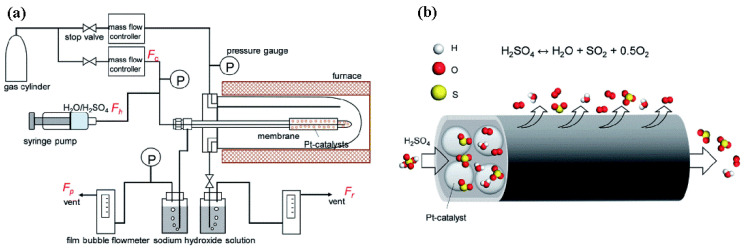
(**a**) Schematic of an experiment for steam and H_2_SO_4_ treatment and gas permeation; (**b**) schematic of the SiC membrane used for H_2_SO_4_ decomposition in a membrane reactor at 600 °C. Reprinted from [34] with permission.

However, under the harsh environment of the sulfuric acid decomposition reaction, the structures of most membrane materials are destroyed, and the permselectivity is lost due to their low thermal stability, oxidation resistance, hydrothermal stability, and chemical resistance. In a recent study reported by the authors’ group [34], a SiC membrane was first explored under extreme conditions (i.e., H_2_SO_4_ vapor at 600 °C). The gas permeance of the SiC membrane hardly changed or decreased slightly after treatment 20 h under H_2_SO_4_ vapor at 600 °C, indicating that the SiC membrane has high chemical stability. Subsequently, the membrane was used in a membrane reactor for H_2_SO_4_ decomposition at 600 °C, as shown in Figure 27. The shift of the equilibrium of the reaction by the timely removal of O_2_ via the membrane was promoted toward the product side. Consequently, the SiC membrane achieved a conversion of 41%, which was much higher than the system without O_2_ extraction (25%). The combination of high separation performance and excellent chemical resistance at high temperatures suggests that SiC membranes are the most promising candidates for membrane reactors for H_2_SO_4_ decomposition.

## 5. Conclusions and Outlook

Considerable research has been conducted on the development and application of SiC membranes. Commercially available SiC membranes have been used in separation processes for liquid media, such as water treatment and desalination. These SiC membranes are well known in industry as UF and MF membranes. Research on developing and applying SiC membranes for gas separation has rapidly expanded. This review, for the first time, summarizes the progress of SiC membranes for gas separation while focusing on the preparation routes for SiC membranes and their application. The preparation approaches for separation layers of SiC membranes include CVD/CVI deposition and pyrolysis of the polymeric precursor. Generally, SiC membranes formed using the CVD/CVI deposition route have dense structures that result in low permeability, which makes such membranes suitable for small-molecule gas separation. However, the pyrolysis of a polymeric precursor is the most common and promising preparation route for SiC functional ceramics and membranes. This method includes precursor selection, coating/shaping, curing for cross-linking, and pyrolysis. Among these steps, the precursor, curing method, and pyrolysis temperature affect the final microstructure and separation performance of the resulting membranes. Based on the discussion of these influencing factors, there is now a good understanding of the evolution of membrane microstructures and how to control membrane microstructures according to the application purpose.

In addition, the thermal stability, oxidation resistance, hydrothermal stability, and chemical resistance of SiC membranes are major advantages. These factors are affected mainly by membrane preparation conditions or precursor properties (including modification). In particular, the stability of membrane operation at moderate to high temperatures in the presence of steam is a notable concern. Although certain stabilities of SiC membranes degrade slightly under certain severe conditions, they maintain high separation performance. SiC membranes are the most promising candidates for high-temperature separation, such as membrane reactors owing to their robust advantages and high separation performance. This review aims to provide meaningful insights and guidance for developing SiC membranes and achieving excellent gas separation performance. Additionally, it is necessary to develop other innovative methods for preparing advanced SiC membranes with high performance, and new applications of SiC membranes can be foreseen due to such advancements.

## Figures and Tables

**Table 1:** Advantages and disadvantages of inorganic membranes with amorphous structures.

Membrane Material	Amorphous Structure	Advantages	Disadvantages
Silica (SiO_2_)	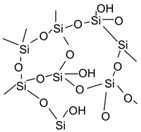	Good thermal stability at moderate temperatures Controllability of pore sizes from the sub-nano to several nanometer range High separation performance for small-sized gases (e.g., H_2_)	Low-level hydrothermal stability Easy sintering at higher temperatures
Carbon molecular sieve (CMS) or carbon membranes	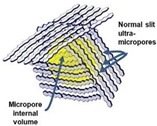 [12]	Excellent chemical stability Rich ultra-micropores Ability to distinguish molecules of almost the same size Surpass the trade-off for gas separation (especially for hydrocarbons)	Instability in the presence of water vapor High cost of polymeric precursors (e.g., polyimide) Limited oxidation resistance (enlarge or destroy the membrane pores) Brittle, difficult to scale-up
Silicon carbide (SiC)	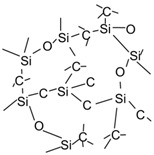	Have excellent chemical, mechanical, and hydrothermal stability; high oxidation resistance Simple fabrication process; easy to scale-up Membranes with controlled structures Wide range of applications for filters (MF, UF, NF, and RO) and gas separation	Multiple-layer coatings High cost of polymeric precursors

Amorphous structure of CMS adapted from Ref. [12] with permission.

**Table 2:** Fabrication process and properties of representative SiC membrane filtrations (MF and UF).

Shape and Pore Size of Supports	Raw Materials of Layers on Supports		Layer Deposition Method	Top Layer Thickness [μm]	Thermal Treatment	Pore Size (Type: MF, UF)	Applications and Other Remarks	Ref.
	Transition Layer	Top Layer						
Disk, 15 μm	na.	α-SiC powder (10 μm), SiC whisker, methylcellulose (MC) ^2^, CaO ^5^, ZrO_2_^5^, mullite ^5^, TL-56NQ ^4^, water ^1^	Spray coating	125	1150–1250 °C, 2 h in air; then 1350–1500 °C, 4 h in Ar	2.31 μm (MF)	Hot gas filtration (high permeability, dust removal efficiency, 99.95%) Excellent corrosion resistance in both H_2_SO_4_ and NaOH Excellent thermal shock resistance	[48]
Flat tube, 1.8 μm	na.	SiC powder (0.55 μm), IPA ^1^, PVA ^3^, PEG ^3^, Darvan-CN ^2^, water ^1^	Dip-coating	12–30	900–1300 °C, 1 h	75–155 nm (MF)	Water filtration (high purity water permeance) Crack-free oxidation-bonded SiC membranes	[49]
Flat tube, 34.92 μm	na.	SiC powder (22 µm), B_4_C ^5^, PVA ^3^, TMAOH ^2^, water	Dip-coating	~100	2200–2250 °C	9.93 μm (MF)	Gas filtration SiC membranes prepared by co-sintering process Good mechanical properties	[50]
Tube, 15 μm	na.	α-SiC powder (0.4 μm and 0.6 μm), Al(NO_3_)_3_·9H_2_O ^5^, Optapix CS-76 ^3^, polysaccharide dicarbonic acid polymer ^3^, water ^1^	Dip-coating	27.3–29.4	1600–1900 °C	0.35 μm (MF)	Oily wastewater treatment (remarkable separation performance) Multi-channeled tubular membranes High chemical and mechanical stability	[51]
Flat sheet, 5.6–14.1 μm	na.	SiC powders (0.5 µm and 3 µm), PAA ^2^, CMC ^3^, water ^1^	Dip-coating	60	1900–2000 °C in vacuum	0.5 μm (1900 °C); 1.4 μm (2000 °C) (MF)	Gas filtration Homogeneous structure	[52]
Flat disk, <100 μm	Consisting of several SiC layers; pore diameter <300 nm	α-SiC powder (0.4 µm), AHPCS ^6^, hexane ^1^, hexane/tetradecane ^1^	Dip-coating	10–19	200 °C, 1 h; 400 °C, 1 h; and then 750 °C, 2 h	<50 nm (UF)	Water filtration Nearly defect-free SiC membrane for UF applications Complex pore structure	[53]
Tube/na.	SiC powder (0.6 µm), acetone; pore diameter =130 nm	PS ^7^, toluene ^1^, AHPCS ^6^, hexane ^1^	Slip-casting + dip-coating	7	200 °C, 1 h, 400 °C, 1 h, and then 750 °C, 2 h in Ar; 450 °C, 2 h in air	Nanoporous SiC membranes	Gas separation Polystyrene sacrificial interlayers Improved membrane performance due to the sacrificial interlayers	[54]

Superscript notation: 1 = solvent; 2 = dispersant; 3 = binder; 4 = defoaming agent; 5 = sintering additive; 6 = polymeric precursor; 7 = pore former.

**Table 3:** Representative processing parameters of SiC membranes obtained by CVD/CVI techniques.

Membranes	Precursor	Supports	Deposition Temperature	Ref.
SiCN	SiH_4_/C_2_H_2_/NH_3_	α-Al_2_O_3_; disk	1050 °C in Ar	[60]
SiCO	SiH_2_Cl_2_/C_2_H_2_/H_2_	γ-Al_2_O_3_/α-Al_2_O_3_; tube	800–900 °C in H_2_	[61,62]
SiC	Triisopropylsilane (TPS)	SiC; disk; and tube	760–800 °C in Ar/He	[59]
SiC	Silacyclobutane (SCB)	Ni-γ-Al_2_O_3_/α-Al_2_O_3_; tube	515 °C in Ar	[63]
SiC	1,3-disilabutane (DSB); TPS	α-Al_2_O_3_; γ-Al_2_O_3_/α-Al_2_O_3_; tube	TPS: 700–800 °C in He, and annealed at 1000 °C; DSB: 650–750 °C in He	[64,65]

**Table 4:** Various (potential) applications of SiC membrane for gas separation.

Gas Separation	Applications (or Potential Applications)
H_2_/N_2_	Ammonia purge gas
H_2_/hydrocarbon	Refinery hydrogen recovery, alkane dehydrogenation
H_2_/H_2_O, H_2_/CO_2_, H_2_O/CO	H_2_ production, water–gas shift, thermochemical water splitting
He/hydrocarbon	Helium separation
He/N_2_	Helium recovery
O_2_/SO_2_, O_2_/SO_3_	O_2_ separation in H_2_SO_4_ decomposition
CO_2_/(hydrocarbon or N_2_)	Acid gas treatment, greenhouse gas capture
H_2_O/Air	Air dehydration

**Table 5:** BET surface area and micropore volume based on nitrogen adsorption for the PCS powders and TiPCS powders as a function of pyrolysis temperature (all powders were air cured at 200 °C for 2 h and pyrolyzed under the same conditions).

Pyrolysis Temperature [°C]	BET Surface Area * [m^2^/g]		Micropore Volume ^#^ [cm^3^/g]	
	PCS [72]	TiPCS [82]	PCS [72]	TiPCS [82]
350	449	dense polymer	0.2164	dense polymer
550	520	66	0.2326	0.02
650	316	498	0.1304	0.174
750	0.754	245.7	0	0.084
800	—	143.1	—	0.046
1000	—	0.4	—	—

* BET surface area was obtained from the linear BET plots over the range of 0.001 < *P/P*_o_ < 0.15. ^#^ The micropore volume was calculated using *t*-plot analysis.

## Data Availability

Not applicable.
